# Postdigital Applied Systems Science Education: Toward an Integral Framework, Curriculum, and Pedagogy

**DOI:** 10.1007/s42438-022-00305-4

**Published:** 2022-03-29

**Authors:** Michael Hogan, Owen Harney

**Affiliations:** National University of Ireland, Galway, Ireland

**Keywords:** Systems science, Postdigital, Education, Collective intelligence, Teamwork, Sustainable wellbeing

## Abstract

The design of systems to support sustainable wellbeing is contingent upon lifespan education of *Homo sapiens* and ongoing efforts to cultivate individual and collective intelligence. The Postdigital Applied Systems Science Education (PASSE) framework presented in this paper highlights the need for greater investment in educational infrastructures that support the development of collective intelligence, teamwork, and system design skills. We propose that the implementation of PASSE involves group- and project-based work focused on developing (1) an understanding of systems, (2) an understanding of group dynamics relevant to the management and design of systems, and (3) skill in the application of applied systems science methods that can be used by groups in the management and redesign of systems. To showcase curricular and pedagogical challenges and opportunities, we describe key features of our current delivery of PASSE along with future plans and prospects. Aligned with postdigital perspectives and innovations at the nexus of biology, information, and society, we highlight the potential for ongoing redesign of educational infrastructures and technologies that enhance societal teamwork and system design capabilities that allow us to address increasingly complex societal challenges.

## Introduction

*Homo sapiens* operate individually and collectively as living systems that seek to survive, adapt, and flourish as part of larger world systems. As the complexity of human and world systems has increased (Pettersson 1996), resilience and sustainability are increasingly under threat (Falk et al. 2021). As a group-living species, human adaptive success in the modern world is contingent upon education, which facilitates both individual and collective intelligence and the dissemination of knowledge and skill needed to manage systems. Notwithstanding threats to sustainability, technology and science continue to develop and innovative approaches to education are emerging which push the limits of our evolved biological constraints as a species. This includes the technologies, tools, and methods that support systems thinking and collective action capabilities. Postdigital perspectives and innovations operating at the nexus of biology, information, and society are central to future collective adaptations in this context (Jandrić 2021).

The postdigital operates both as a critique of the digital as a technological fix (Peters and Besley 2019) and as a philosophical and design-oriented perspective that highlights challenges and possibilities linked to developments in bioinformatics and broader convergences arising from the Nano-Bio-Info-Cogno paradigm which has been central to science and technology innovation in recent years (Peters 2020; Peters et al. 2021a). Central to postdigital enquiry is the development of a critical philosophy that examines the nature of these convergences and ways in which bioinformatic, socio-technical, educational, economic, and political systems could transform how *Homo sapiens* live and interact with one another and their environment.

By focusing on future possibilities, the postdigital also orients enquiry to issues of evolutionary advance and change, and the ethical and design challenges that need to be considered. It is speculated that Nano-Bio-Info-Cogno convergences may go through many iterative cycles in the next decades, allowing for new forms of genetic-digital intelligence and potentially make possible a form of bioeconomy that is environmentally self-renewing, which is significant in light of current sustainability challenges and the prospect of mass extinction (Peters et al. 2021a). However, these are idealistic speculations and a parallel line of enquiry seeks to understand the educational and collective intelligence requirements that support ethical system designs.

As such, consideration of ethical issues linked to the future sustainable wellbeing of *Homo sapiens* is central to postdigital enquiry, as is consideration of educational system design requirements that will be important in shaping foundational knowledge and skills that influence future Nano-Bio-Info-Cogno and related socio-technical, economic, political, and environmental system designs. For example, at a fundamental level, postdigital scholars have noted that the definition of literacy in language, digital skills, and data may require new hybrid concepts and the design of new educational and socio-political terminology, training infrastructures, and practices that support citizen literacy (Peters et al. 2021b). More generally, consideration of ethical issues related to the meaning of sustainable wellbeing also implies ongoing collaborative learning and a design-oriented and experimental ethos and practice that support learning together. In this sense, postdigital education is oriented to enquiry in relation to collective intelligence (CI) and the design of CI infrastructures.

Questions arising as a consequence of new convergences across the Nano-Bio-Info-Cogno paradigm include how we understand life evolving and how we collaborate in the experimental design and development of new artificial intelligence (AI) and algorithmic systems that become increasingly integrated with human biological systems (Peters et al. 2021a, c). This collaborative enquiry and design-oriented effort extend to questions about how we co-exist, survive, adapt, and potentially flourish in relationship with other life forms (Peters et al. 2021b). The significance of these questions has been starkly revealed in the recent example of the COVID-19 pandemic, where *Homo sapiens’* collective relationship with one another and their environment has been fundamentally transformed, requiring new levels of solidarity and CI design that has tested the limits of our cooperative capacities (Hogan 2020). From an educational perspective, Jandrić and Ford (2020) highlight the significance of recognizing this broader ecological, relational, and design challenge.

A central tenet of the Postdigital Applied Systems Science Education (PASSE) framework presented in this paper is that the management and redesign of systems in the modern world requires greater investment in CI infrastructures such that groups can work well together to promote survival, adaptation, and flourishing. This includes an educational focus on the training and development of CI competencies and systems thinking and collective action capabilities, the facilitation of which can be supported by systems thinking tools, a curriculum focused on systems and transdisciplinary system design, and project-based pedagogical approach focused on team learning.

Notably, when it comes to the design of CI infrastructures, Mulgan (2018) argues that conscious orchestration of CI is needed—we need to design for CI in a disciplined and careful way. Mulgan notes that if we fail to establish a discipline of CI—in schools, universities, governments—there is a risk that our biased, over-confident, manipulative, competitive, groupish tendencies will play out in more powerful and damaging ways across a range of technologically innovative, but poorly conceived, assemblies that dot the intelligence design landscape.

PASSE provides a design- and practice-oriented framework that emphasises a curricular and pedagogical focus on the collective, group, and team structures and processes that are central to the operation of a viable CI and applied systems science. We highlight as problematic the predominant focus on individuals and the limited exposure to collaborative systems thinking and systems design methodologies in educational settings (Hogan et al. 2014, 2015b). We argue that in order to implement PASSE in practice, educators and students need to cultivate (1) an understanding of systems (Hogan et al. 2018; Hogan 2020), (2) an understanding of group dynamics relevant to the management and design of systems (Hogan et al. 2021), and (3) skill in the application of applied systems science methods that can be used by groups in the management and redesign of systems (Hogan et al. 2017). While (1) and (2) imply a specific curriculum focus, (3) implies a pedagogical approach oriented toward teamwork and collaborative use of tools and methods for systems thinking and system design work.

Central to PASSE is a focus on the role of CI facilitators and developing key CI facilitation competencies needed to support groups in the use of tools and methods for system design work (Broome and Hogan 2021). While the use of technology and specific tools and methods is central to modern systems science, including recent innovations in the use of systems thinking tools in online CI facilitation contexts (Hogan et al. 2022), in this paper, we argue that a postdigital approach (Jandrić et al. 2018; Knox 2019) to applied systems science education needs to be integral, in the sense that it embraces a holistic systems perspective. For example, a holistic systems perspective is important in relation to timescales in the analysis and operation of living systems, and (inter)subjective and (inter)objective dynamics that need to be understood in the design and management of systems.

Furthermore, a postdigital and design-oriented approach to PASSE must envision how innovation in socio-technical infrastructures can help to advance the delivery and real-world applications of applied systems science, while also reinforcing teamwork and the empowerment of ethical CI design focused on designs for sustainable wellbeing. We argue for the use of a design-based research (DBR) approach to future iterations of PASSE curriculum and pedagogy.

## Educational Context

PASSE is situated in a context of ongoing educational transformation, including increased global uptake of third-level education, ongoing educational technology innovation, increased emphasis on interdisciplinary research and teaching, and a move toward project- and problem-based teaching and learning (Altbach, Reisberg, and Rumbley 2019). While a team-based approach to science and technology innovation is increasingly prevalent, this has not been coupled with a strong focus on education for teamwork or the development of a discipline of CI (Mulgan 2018; Hogan and Broome 2020). To showcase curricular and pedagogical challenges and opportunities for PASSE, we describe some key features of our current implementation of applied systems science education along with a number of curricular aspirations and future plans and prospects.

We present a summary overview of our current 12-week training module, Introduction to Collaborative Enquiry and Applied Systems Science, along with feedback from students on their learning experiences. Our module has evolved over a 7-year period and is currently offered as an elective to 3rd year undergraduate students at NUI, Galway, Ireland. While all students taking our module study psychology, they also have a variety of second-degree subjects (e.g. Information Technology, Geography, Sociology, Political Studies, and Economics).

Our module was inspired by the work of John Warfield, past president of the international society for systems. Notably, Warfield (2010), in his *Horizon College* proposal, envisioned students from various disciplines learning together, and bringing their knowledge and experience together in a process of collaborative system thinking and design work. For the purpose of project work maximizing small group team dynamics, students attending our module (*N* = 25) are divided into four or five groups, each of which includes 5 or 6 students. An effort is made to include a diversity of second-subject expertise across each project team. To provide sufficiently intensive instructional support and facilitation across the four or five teams, the module is co-delivered by two instructors, both of whom have worked on multiple local, national, and international CI system design projects.

Students taking our module engage in project work aligned with ongoing national and international projects being undertaken by the Collective Intelligence Network Support Unit (CINSU) at NUI, Galway. Student teamwork involves cooperative reading and the use of systems thinking tools to support understanding of a specific applied problem, along with the use of scenario-based design methods to support the development of a socio-technical solution. Systems thinking products and socio-technical design solutions are shared across teams in a series of group presentations in the final week of the module. Previous student projects have focused, for example on the design of technology-supported systems to promote transparent, open democracy and government; the design of personalised nutrition services for older adults; and the design of technology-supported systems to promote adherence to contraception among university students*.* Over the past 2 years, in part as a response to the global challenges presented by COVID-19 (Hogan 2020), we have sought to incorporate a broader curricular perspective, which we introduce in this paper as central to our PASSE framework.

## Cultivating an Understanding of Systems: Integral Systems Thinking as a Foundation for PASSE

As part of educational training, it is useful to foster an integral perspective in relation to systems (Meadows 2008). Understanding systems is a central pillar of PASSE, and a broad understanding of systems quickly reveals to educators and students the significance of group dynamics and the importance of CI in the management and redesign of systems (Ostrom 2009). Working together to understand, design, and manage systems is a central pedagogical focus of PASSE.

**Fig. 1:**
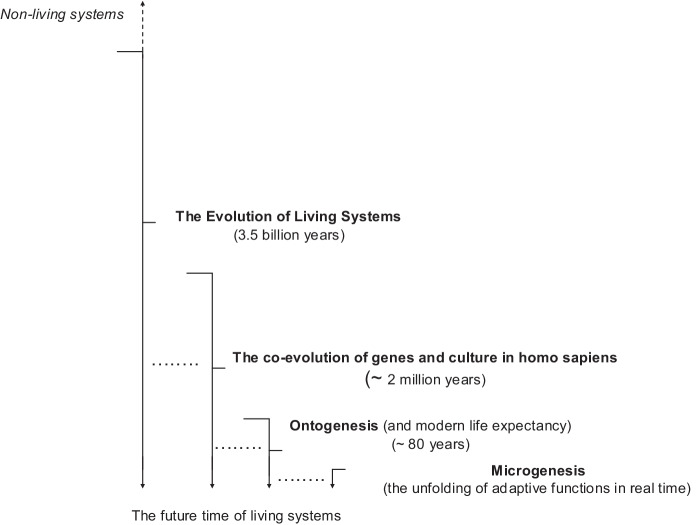
Nested time scales in the analysis of living systems

In a classroom context, it is useful to begin with a shared focus on living systems and the issues of survival, adaptation, resilience, and sustainability, specifically, by reference to the inter-objective world as it manifests across four nested time scales in the analysis of living systems (Fig. 1). Pedagogically, as we move toward collaborative systems thinking, it is valuable to adopt a transdisciplinary lens of enquiry to import knowledge from relevant disciplines, which can help students and educators to reflect upon and develop a deeper dialogical understanding of systems. Consider four nested timescales in the analysis of living systems: (1) the evolution of living systems (~ 3.5 billion years); (2) the co-evolution of genes and culture in *Homo sapiens* (~ 2 million years); (3) ontogenesis (i.e. average modern life expectancy and lifespan, ~ 80 years); and (4) microgenesis (i.e. the unfolding of adaptive functions in real time).

### Living Systems

The broadest timescale of analysis is the period within which living systems have been evolving, circa 3.5 billion years (De Duve 2002). An eye to the long history of living systems can enhance our general understanding of survival, adaptation, and flourishing. It can open our awareness to the intimate relationship between living systems and their environment, the common features of life evolving, the range of activities pursued by different species, and the actions that *Homo sapiens* are uniquely capable of. An eye to the long history of living systems can also help us to appreciate why resilience and sustainability are basic notions that apply to all living systems, and ecosystems, and, understandably, why resilience and sustainability emerge as common themes across multiple scientific disciplines.

### Gene-Culture Co-evolution

Gene-culture co-evolution is the process whereby information that has been transmitted from generation to generation has altered biological systems (Richerson and Boyd 2005). Cultural evolution—specifically, the emergence of new ideas, values, skills, tools, and artefacts of culture—can be viewed as essential to advancing our CI skills and our ability to work well together in teams. PASSE highlights how cultural evolution is driven in part by imitation, conformity, and a tendency to follow ‘successful’ members of any given cultural group (Boyd and Richerson 2005). Across the broad fields of science, technology, and governance, inter-group conflict (Tropp and Tropp 2012) is an evolutionary given that influences the broader dynamics of cultural evolution. At the same time, conflict and cooperation co-exist and both are essential to the ‘creative’ potential of living systems (Belussi and Orsi 2016).

### Ontogenesis

Ontogenesis or lifespan development (Baltes et al. 1998) is a central focus in fields such as developmental psychology, education, and the learning sciences. PASSE highlights a problem that, currently, the discipline of CI tends to ignore ontogenetic and related pedagogical considerations (Malone 2018; Mulgan 2018). The explicit development or ontogenesis of CI (much like the development of individual intelligence) requires an explicit educational training focus (Hogan et al. 2015a, b). The sciences focused on ontogenesis—most notably, developmental psychology—are relevant here. They reveal to students, educators, and practitioners the importance of ontogenetic considerations in efforts to coordinate the intelligence of individuals in a group or team setting (Fischer and Bidell 2006). A broad variety of literacy, numeracy, graphicacy, tool, and interpersonal skills are needed to support successful adaption in modern human environments. Also, component operations supporting individual intelligence and CI (Mulgan 2018)—perception, attention, memory, reasoning, problem solving—are acquired over months and years, and their coordination within and across individuals takes time.

### Microgenesis

Analysing behaviour as it unfolds in real time—over seconds, minutes, hours, days—is very important for understanding the *ongoing* state of human systems (Fischer and Bidell 2006). The dynamics of behaviour as it unfolds in real-time is the essential material of sustainable cooperative groups and high functioning teams—it is the essential glue that supports problem solving and resilience and sustainable well-being in groups. The microgenetic lens of enquiry is important for understanding methodological skill development and application of CI and systems thinking methods in PASSE. By engaging in collaborative systems thinking and socio-technical design work, students and educators can begin to understand acts of perspective-taking, knowledge exchange, reasoning, decision-making, and learning in groups and teams, and they can develop skills that support optimisation of individual and collective actions supporting the application of different systems thinking and systems design methods.

## Pedagogical Coordination Across (Inter)Objective and (Inter)Subjective Worlds

**Fig. 2:**
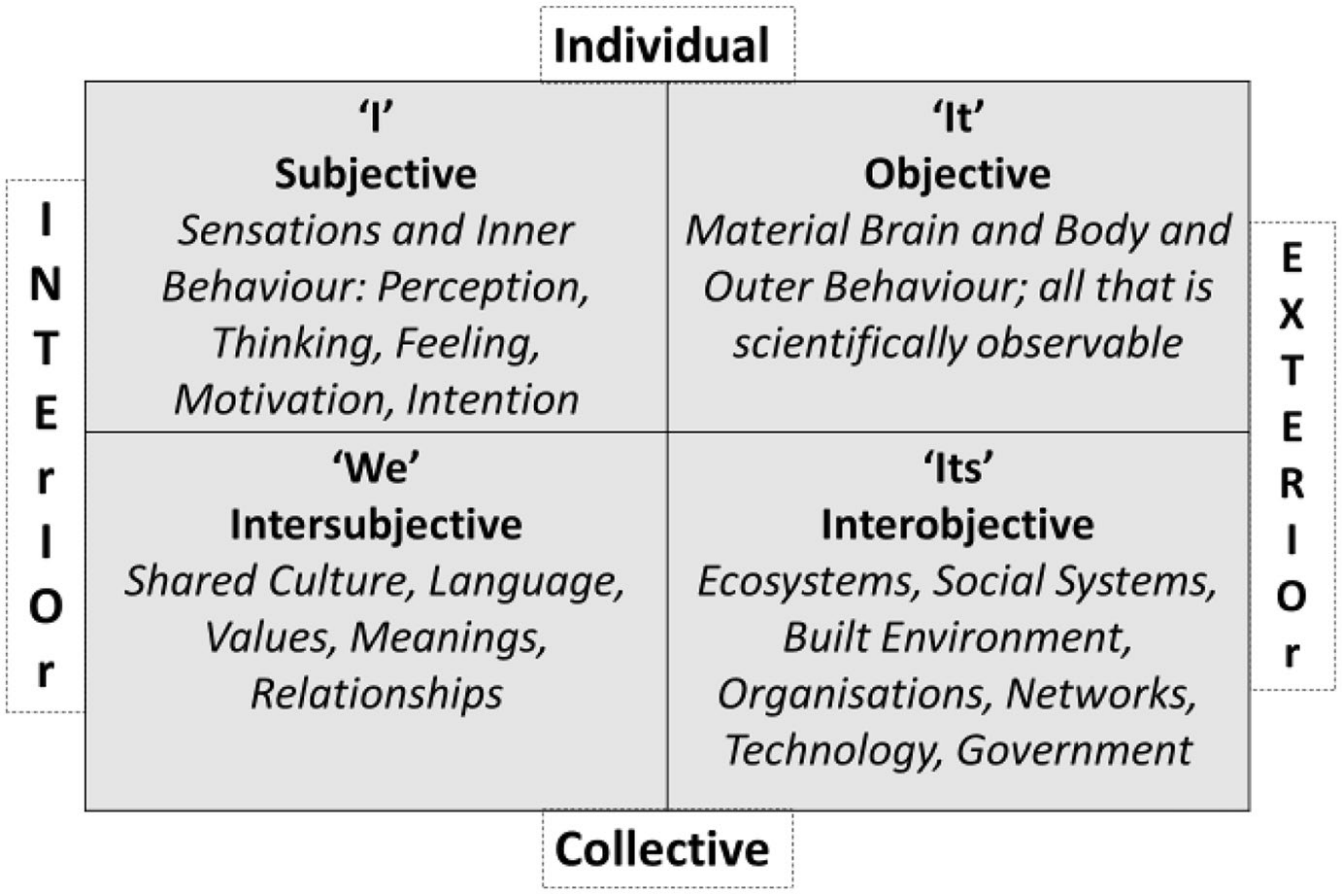
Perspective taking across four worlds

As part of an integral perspective, PASSE highlights the value of coordinating understanding of systems across timescales of analysis with understanding of exchanges that occur within and across the four *worlds* characterised by Wilber (1997): the I-It-We-Its Worlds (Fig. 2). Each world highlights a unique lens of enquiry in the analysis of systems.

Exchanges across the I-It-We-Its Worlds are ever-present and naturally play out in the micorgenetic and ontogenetic context of CI and system design work. First, we have the private exchanges occurring within individuals, in a subjective world (the I-World in the upper left quadrant of Fig. 2). Systems thinking at the group level requires communication *between* individuals, but it is challenging to express all the thoughts and feelings that emerge from *within* our subjective I-World (Hogan 2020). We can use tools to help structure and communicate our thinking and reasoning in a group context (Dwyer et al. 2010, 2012), but facilitating CI and systems thinking at the group level is always challenging as it involves making the private world public, in a way that supports group problem solving (Harney et al. 2015; Harney et al. 2012; Harney et al. 2017).

Supporting CI and systems thinking requires the coordination and facilitation skills of CI group facilitators (Broome and Hogan 2020; Hogan and Broome 2020, 2021). While facilitating the group through the various steps in the CI and systems thinking and design method the CI facilitator plays an important role in allowing the I-World to manifest objectively as part of the It-World (i.e. in the actions of individuals), where it can be further coordinated in the actions, operations, and designs of the Its-World (e.g. in the coordinated actions of design teams).

Individual actions are observed in the It-World in the upper right-hand quadrant in Fig. 2. For example, when a student in the classroom talks, writes, draws, or computes using numbers, signs, or symbols, the teacher and other students in the group can perceive and interpret the act, and further coordinate and combine actions to support learning, skill development, or the design and production of specific artefacts. At the same time, a teacher or CI facilitator working to support systems thinking in a group also works in the context of an intersubjective world (We-World, lower left quadrant in Fig. 2), where the thinking and feeling of one student relates to the thinking and feeling of other students. The We-World includes the common (and varied) expectations, values, norms, ideas, and knowledge that groups shed light on when they talk or behave in a social setting. Again, revealing the We-World to the group requires skilled facilitation and in the context of collaborative systems thinking and systems design work, this skilled facilitation and navigation of We-World dynamics is critical for consensus-building, cooperation, teamwork, and productive group dynamics.

As applied systems thinking is oriented to a shared problem situation that manifests in a shared environment, groups will naturally orient themselves to the measurable dynamics of the inter-objective world (Its-World). Indeed, in developing a foundational understanding of systems, students will come to appreciate that Its-World problems are ever-present and open to shared observation—political polarisation, conflict, war, crime, poverty, environmental degradation, pandemics, chronic disease, mental illness, social disengagement, inequality, and countless others. When a group is working to address a societal problem, regardless of the local or global scale of the problem, they usually want access to measurable inter-objective phenomena, but they also need to arrive at some consensus as to which aspects of the inter-objective world are most relevant to the problem being addressed.

As soon as group members make individual and collective judgements as regards what is most relevant or important to focus on, their interior I-Worlds and We-Worlds begin to select from, place a structure on, and shape the design of Its-World phenomena. Again, the role of the CI facilitator is critical here, as they support idea generation, selection, and structuring through the application of transparent methods. The facilitators support clarity and shared understanding in the communication of ideas and facts, the production of an audit trail of all ideas generated, and the implementation of voting and idea structuring methods.

Although our current implementation of PASSE involves the application of a specific set of tools and methods that students learn to use as they develop skill in CI facilitation, the scope of tools used in an idealised PASSE framework is broader. Jackson (2019), in *Critical Systems Thinking and the Management of Complexity*, showcases a range of systems thinking and design methods for working in the context of technical complexity (e.g. Operations Research), process complexity (e.g. the Vanguard Method), structural complexity (e.g. System Dynamics), organisational complexity (e.g. Organisational Cybernetics), people complexity (e.g. Interactive Planning), and coercive complexity (e.g. Team Syntegrity).

PASSE recognises that our movement into the future unfolds across all four worlds, the I-It-We-Its worlds, and students learn that we occupy a vast subjective and objective space that is deeply interconnected. By fostering an integral perspective on systems that allows for reflection on the temporal, (inter)objective, (inter)subjective dimensionality of systems, students can begin to engage in collaborative problem-based learning grounded in an appreciation of the *totalities* inherent in all system design projects.

**Fig. 3:**
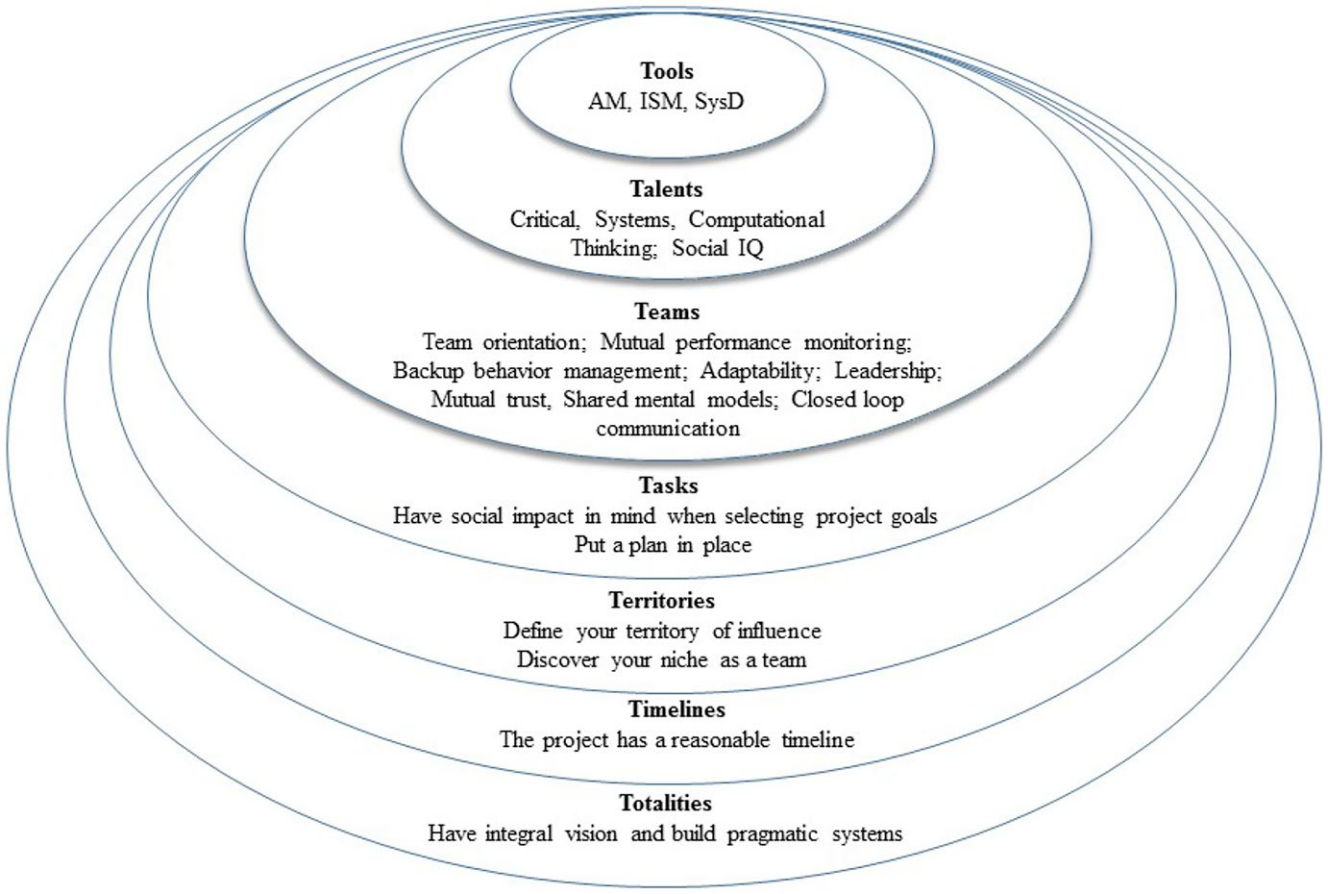
The 7-Ts framework—Building upon the 3-Ts framework of Hogan et al. (2015a, b)

This focus on *totalities* and an integral perspective in relation to systems informed some of our previous design thinking in relation to applied systems science education. In particular, members of our PASSE design team previously envisioned systems science education as involving a core pedagogical focus on (1) *tools*, (2) *talents*, and (3) *teams*, i.e. the 3-Ts represented in the three central circles in Fig. 3 (see Hogan et al. 2015a, b). These aspects of systems science education were seen at the time to be embedded in a broader framework including a pedagogical focus on (4) project *tasks*, (5) *territories* of application, (6) *timelines* of project work, and (7) *totalities* of perspective in relation to the focus of work.

At the time of publication, Hogan et al. (2015a, b) focused only on the 3-Ts framework. PASSE highlights a revised approach to curriculum delivery and also revisits the earlier 7-Ts framework envisioned at the time. In particular, PASSE highlights the pedagogical value of focusing first on integral systems thinking and perspective-taking in relation to pragmatic challenges and constrains of system design, before moving to specific project tasks and applications. By reference to the 7-Ts framework in Fig. 3, in a project-based curriculum context, students transition from a focus on *totalities* (i.e. integral systems thinking and perspective taking) to a focus on applied systems science projects that have a specific *timeline*.

These applied projects address the design of systems within specific *territories* or niche areas of application. This implies teamwork oriented around specific *tasks.* Central to the current design of our PASSE module, as described further below, are a clear set of task steps that students need to follow in the applied system design process. With this task and project focus, PASSE then orients students to the significance of *teams* in the design of systems, and the importance of collective facilitation in supporting teamwork activities (Harney et al. 2015; Broome and Hogan 2020). Finally, central to PASSE are the *tools* and *talents* that need to be coordinated by *teams* as they work across different knowledge and skill domains in any system management or design project.

In an educational training context, PASSE proceeds using a project- and team-based pedagogical approach to cultivate relevant talents (e.g. critical, systems, and computation thinking skills and social intelligence), and skilled use of tools and methods (e.g. argument mapping tools, interpretative structural modelling software, and scenario-based design methods) supporting systems thinking and design work.

## Orienting Students to Project- and Team-Based PASSE Using Interactive Management

In this section, we describe our starting point in the development of PASSE, and how we set out in particular to advance John Warfield’s proposal for systems science education (Warfield 2010). While Warfield had a vision for systems science education, which envisioned students from various disciplines bringing their knowledge and experience together in a process of collaborative system thinking and design work, unfortunately, Warfield never had the opportunity to design and deliver an applied systems science education programme.

The 7-Ts framework provided us with an initial starting point as we sought to integrate key tools, talents, and team dynamics central to the design and delivery of an applied systems science module at University level. While a detailed description of the particular tools and methods we use (including procedural detail on Interactive Management and associated software) is provided elsewhere (Hogan et al. 2014, 2015b), we elaborate on some key aspects of our most recent curricular developments and pedagogical approach. We focus in particular on the group dynamics knowledge, CI facilitation, and teamwork skills we seek to cultivate in students. We also present a broader overview of our module, which includes more detail on methods, and we highlight some key areas of ongoing development.

### John Warfield’s Approach

**Fig. 4:**
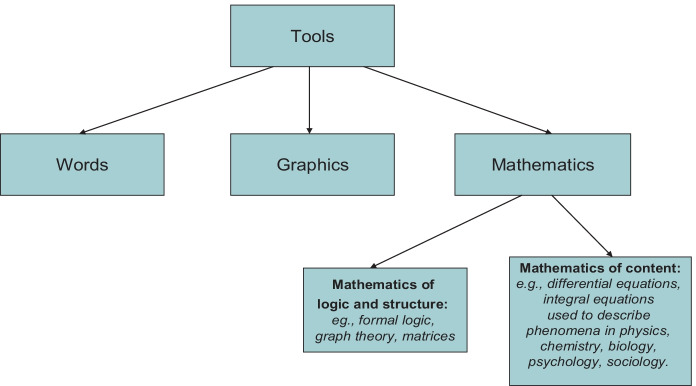
Systems science tools needs to work with our capacity to share meaning using words, represent causality using graphics, and model complexity using mathematics

Warfield (2006) argued that resolving complex scientific and social problems is contingent upon the collective action of groups working with applied systems science methods and tools. Applied systems science will be most effective if it makes use of tools that integrate our capacity to share meaning using words, represent causality using graphics, and model complexity using mathematics (see Fig. 4). In developing his unique applied systems science method, Interactive Management (IM), Warfield integrated all three of these components in its design.

To support groups in developing structural models that describe interdependencies between problems in a systems model, Warfield leveraged the mathematics of logic and structure, drawing in particular on synergies between formal logic, matrices, and graph theory. The method allows the logic of group members, as represented in a matrix of decisions, to be represented visually in a graph (i.e. as a systems model).^1^

When (1) a *team* of people come together to focus on (2) a complex *issue*, they need (3) a *methodology* that helps them achieve an adequate synthesis of knowledge that supports collective understanding and action in response to the issue (Warfield 1976). Warfield highlighted the need to partition the team into three sub-groups:

- Stakeholders—the people who have a stake in the issue being considered.
- Content specialists—the people who have specialized knowledge that is relevant to an issue under consideration.
- Structural modelers—the people whose task it is to structure the issue being considered. (Warfield 1976)

While stakeholders and content specialists communicate the knowledge essential for understanding the issue or problem the team is addressing, the structural modelers facilitate the team in structuring their knowledge using specific facilitation strategies and tools.

PASSE unfolds with a strong focus on building the competencies needed to facilitate teams. In approaching applied systems science project work, students learn that there are a number of other groups that are instrumental in enabling, implementing, and managing the processes essential for complex issue exploration and successful societal problem solving. Warfield (1976) describes three functions in particular—the *enabling* function, the *implementing* function, and the *managing* function—each combining three unique elements and each including people outside of the core systems thinking and design team.

**Fig. 5:**
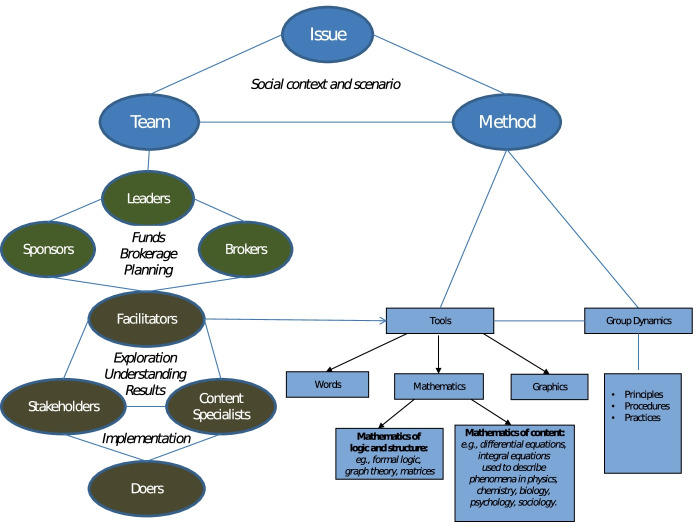
Addressing societal issues using team-based methods. Who is involved and what methods might they use?

- The *enabling* function is critical for any team to proceed with their project work. Tt involves (1) a *sponsor* who controls (2) *funds*, and who has sufficient interest in (3) the *ideas* related to an issue. There is always a cost associated with applied systems science teamwork and other forms of intensive CI work, and a sponsor needs to provide funds to enable any such exploration to proceed.
- The *implementing* function involves coordination between (1) the *stakeholders* in the issue to be explored and (2) the *doers* who decide to act and carry out the proposed actions based on (3) the *results* of exploration.
- Finally, the *managing* function involves (1) *leadership* in identifying issues to focus on and (2) *planning* and designing a scenario for the future, and (3) *brokerage* among the sovereign entities involved, including the sponsor, the facilitators, the stakeholders, and the doers, such that plans that incorporate the results of exploration are translated into results in society (see Fig. 5).

A broad understanding of group dynamics helps system design teams bring together and synthesise the work of content specialists (i.e. those with knowledge of the problem domain) and implementation method specialists (i.e. those with specific skills needed to engineer and implement solutions in the domain). For example, consider projects that seek to mobilize communities (e.g. older adults) to use newly engineered software solutions that allow them to monitor, track, and support their physical and psychological health. In addition to content specialist who understand how physical and psychological health is best supported, we need methodological specialists who know how to mobilize communities, educate individuals, build specific software solutions, create health monitoring tools, etc. When content and methodological specialists work closely with stakeholders (i.e. older adults and their associated family and community networks), this allows the specific issue (i.e. supporting the health of older adults) to be addressed in context.

Increasingly, funding calls for EU and Horizon projects require coordinated action at this level, and PASSE is being developed in response to this need (i.e. to provide the new generation of students with the tools, talents, and teamwork skills that are needed for coordinated project work). In this context, our CI network support unit (CINSU) has been involved in six major EU projects over the past decade,^2^ and in the delivery of our PASSE training to students, we seek to orient students to these EU projects and related CI and design initiatives and projects around the world.

## Group Dynamics and the Facilitation of Applied Systems Science Project Work

**Fig. 6:**
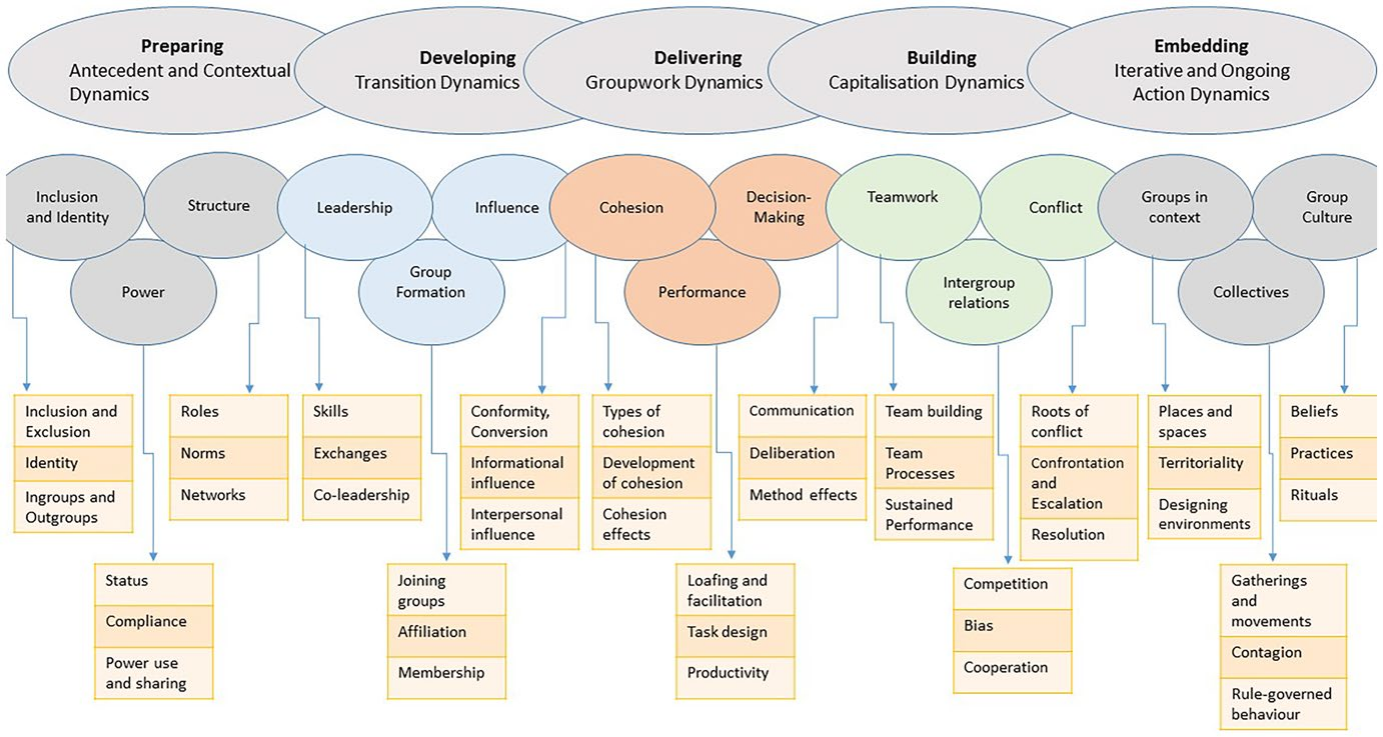
Group dynamics framework for collective intelligence facilitators

Developing an understanding of group dynamics relevant to the management and design of systems is a central pillar of PASSE. The application of applied systems science methods generally involves different phases of work where different group processes are required to advance the systems thinking and collective action of the group. This implies understanding group dynamics that unfold during different phases of working with a group (see Fig. 6). We have proposed a group dynamics curriculum that is broad in focus (Hogan et al. 2021) and draws from relevant scientific literature (Forsyth 2018; Levi and Askay 2020). The curriculum considers group dynamics linked to five different phases of working with a group: (1) *Preparing* **(**Antecedent and Contextual Dynamics); (2) *Developing* (Transition Dynamics); (3) *Delivering* (Groupwork Dynamics); (4) *Building* (Capitalisation Dynamics); and (5) *Embedding* (Iterative and Ongoing Action Dynamics).

### Antecedent and Contextual Dynamics

PASSE highlights first the importance of understanding group dynamics that pertain to what we have called Antecedent and Contextual Dynamics. Notably, in advance of facilitating groups using applied systems methods, we need to understand inclusion and identity and power dynamics and we also need to map and understand the structure of groups we are working with—for example, roles, norms, and network structure—to better understand the context in which we are working. In practice, this generally involves mapping the stakeholder ecosystem, while also working to understand the effects of inclusion and exclusion on group behaviour, the influence of group identity on behaviour, and the dynamics in-group and out-group behaviour. Students also learn about power dynamics, which includes a focus on status, compliance, and power use and sharing within and between sub-groups and individual group members.

### Transition Dynamics

As project work proceeds, there is a focus on Transition Dynamics, including leadership, group formation, and group influence. Students need to understand the role of leaders in supporting collective action capabilities, and the role of leadership skills, leader–follower exchanges, and co-leadership dynamics in project work. As project groups come together, students learn more about group formation dynamics—joining groups, affiliation in groups, and group membership. Given the role of knowledge exchange and deliberation in systems thinking, students also learn about group influence, including conformity and conversion dynamics, and the dynamics of informational and interpersonal influence.

Our PASSE curriculum and pedagogy is project-based. As noted, in its current iteration, four or five teams of 5–6 students use systems thinking tools and methods to address a specific design challenge (e.g. the design of a technology and organisational infrastructure to support adherence to contraception amongst university students). Throughout the systems thinking and design process (which unfolds across weeks 7–12 in the semester-long module) (see Table 1), students acquire group facilitation skills and they come to understand the primary process role of CI and systems design facilitators and the importance of Groupwork Dynamics.

**Table 1:** Week-by-week module focus for Introduction to Collaborative Enquiry and Applied Systems Science

**Week**	**Topic**	**Task**	**Knowledge and skills**	**Tools and methods**
**1**	Introduction to module	- Introductions - Learning outcomes - Module overview - Reflections on dialogue - Tower building task - Attributes of effective collaboration	Collaboration	Tower building Dialogue
**2**	From Collaboration to Collective Intelligence	Present 7Ts model Develop peer monitoring exercise	Introduction to Systems, Group Dynamics, & Teamwork	Question asking Facilitation of dialogue
**3**	Argument Mapping	Argument mapping Reflective log	Critical Thinking	Rationale
**4**	Peer feedback and prompting; Interactive Management	Argument mapping using peer to peer prompts Mock IM session	Systems Thinking Peer feedback	ISM Graphic organiser for peer-to-peer prompts
**5**	Scenario-based design and User Stories	Use scenario-based design to design a mock interface for assisting authors in designing children’s e-books	Design Thinking Collaboration	Scenario-based design and user stories
**6**	Scenario-based design presentations Group projects begin	Present design ideas Decide on topical focus for group project	Collaboration	
**7**	Cooperative reading Idea generation	Engage in literature review Share key points Generate barriers relevant to topical focus	Critical Thinking	Ideawriting
**8**	Cooperative reading Idea generation (cont’d)	Continue literature review and idea generation Develop comprehensive set of barriers	Collaboration Critical Thinking	Ideawriting
**9**	Categorisation Prioritisation Begin systems model-building	Arrange barriers into categories of conceptually similar ideas Vote on key barriers Begin structuring	Critical Thinking Systems Thinking	Paired comparison method ISM
**10**	Systems Model-Building (cont’d), and generation of options	Complete structuring Begin generating options for overcoming barriers	Critical Thinking Systems Thinking	ISM Ideawriting
**11**	Scenario-based design and user stories		Requirement specification Design Thinking	Scenario-based design Design drawing and mock-up
**12**	Present projects		Design Reflection	

### Groupwork Dynamics

In real-world applied contexts, Groupwork Dynamics involve interactions between stakeholders, experts, leaders, and implementation team members. When working with a team, group process facilitation centres of issues of group cohesion, performance, and decision-making. PASSE develops in students an understanding of different types of cohesion dynamics—emotional, task, structural, social, collective—and cohesion-performance dynamics. Students learn about the conditions under which groupwork can facilitate or inhibit performance, and how groups perform under different task structures. Maximizing productivity, including creative and critical thinking outputs during different phases of group work, requires an understanding of the conditions under which different thinking processes are optimized in a group.

In relation to decision-making, students can learn about the importance of communication, including types of talk, information sharing, bias and heuristics, transactional memory dynamics, risky shifts, polarization, and groupthink dynamics. Students can also learn about method effects, in particular, how the specific rules and procedures of different decision-making methods can influence decision-making and collective action.

### Capitalisation Dynamics

As part of Capitalisation Dynamics, students learn more about teambuilding and key team processes, including action processes (e.g. system and goal monitoring, team monitoring, backup behaviour, coordination) and interpersonal processes (e.g. motivation and confidence building, affect management, conflict management) that are central to project work. Students learn how teams build upon their systems thinking and design product outputs in ongoing efforts to implement proposed system designs and changes. This includes learning about cooperative incentives, mutual accountability, and sustained communication and coordination dynamics that are central to ongoing capitalisation and performance in teams. As students become trained as group facilitators, they can learn about conflict and resolution dynamics, which involve a move toward negotiation, understanding, mutual concern, conciliation and forgiveness, and ongoing conflict management.

### Iterative and Ongoing Action Dynamics

PASSE introduces students to Iterative and Ongoing Action Dynamics, which can be seen on the far right of Fig. 6. This includes learning about groups in context, including how places and spaces influence group action and how, for example small changes in places and spaces (e.g. perceived pleasantness, safety and controllability, noise, crowding, temperature, overload and complexity, furniture and seating arrangement) can influence group behaviour. Territoriality is also identified as relevant to organisational dynamics (i.e. ways in which groups claim, mark, and become attached to particular places and spaces, and defend against intrusion by others). Students also learn that cooperative and productive groupwork requires a focus on designing environments, which includes the design of flexible spaces for meetings, seclusion, and for creative work.

More broadly, there is a focus on the dynamics of collectives and group culture. Collectives can generate rapid unfolding dynamics linked to gatherings, crowds, mobs, and panics, and these dynamics are important to understand, as are the dynamics of diffusions (rumors, mass delusions), trends (fads, crazes), and organised social movements. CI efforts that make use of applied systems science methods can be moderated and potentially destabilised by collective dynamics. Also important for students to understand as they grow and develop as group facilitators are the structural and rule-guided collective behaviour dynamics of organisations, which may take considerable time for facilitators to understand in their efforts to support teams they are working with.

Finally, students must learn that group facilitation by its very nature involves immersion in group culture. This implies understanding the beliefs that shape systems thinking and collective action planning, and the practices and rituals of groups.

## Developing Skill in the Application of Applied Systems Science Methods and the Role of the CI Group Facilitator

Facilitating groups using applied systems design methods requires an understanding of the role of the CI facilitator. Although the facilitator is not responsible for content input, the process facilitation is central to groups producing valuable products (Hogan et al. 2014; Hogan and Broome 2021). The facilitators work in the following ways:

1. *Developing a collaborative working relationship with the group.* Engage with sponsors, brokers, and leaders to clarify the context and specific problem or issue the system design group are addressing; clarify the goals of the session; identify and select participants who will take part in the session; serve as a teacher/educator in explaining to sponsors/brokers/leaders what can/will be done and how much time will be required.
2. *Planning appropriate group processes.* Develop a detailed session plan and materials for systems design workshops; select the key systems thinking and system design methodologies and the sequence of activities that will be carried out during and after the session; discuss and clarify with the broker and/or a representative of the participant group the nature of plans and specific methodologies to see if they are appropriate.
3. *Managing logistics.* Manage travel and accommodation logistics for group members; make arrangements for the meeting room; gather the materials that will be used in the session, ensuring food and drink is available to sustain the group throughout demanding work; advance simulation and ongoing monitoring of detail in relation to every aspect of workflow.
4. *Facilitating group process.* Sustain an inclusive and participatory climate throughout systems thinking and design work, and in subsequent follow-up interactions with the implementation team; facilitate communication flows, prevent sessions from becoming platforms for individual presentations, academic debate or political posturing. The group facilitator promotes equality of input in the CI design process, respect for all individual contributions, supporting diverse points of view to be voiced while disallowing premature evaluation. The facilitator prompts participants to listen to and learn from each other and reinforces their commitment to support the work of the group.
5. *Guiding the group to desired outcomes.* System design sessions result in the development of a set of useful design products. Groups can also develop new communication patterns and may build higher levels of trust that carry over into design implementation work, as part of capitalization dynamics. However, the primary focus of the core groupwork phase is to help the group to achieve their key session outcomes. Different products emerge depending on the goals of the session (e.g. a collective vision statement, a detailed plan of action to address a problematic situation, and a systematic set of design requirements for socio-technical solution). The facilitator must keep the group on track toward their desired outcomes by implementing the planned methodologies, while making necessary adjustments in response to the changing group dynamics and changing needs of the group. This may involve interventions such as reminding the group of the context of their work, summarizing and synthesizing progress to date, displaying interim results, redirecting the group back to the task, varying the pace of the work to keep everyone engaged, and bringing the group’s attention to the objectives and goals of the session.

The facilitator role requires sustained curiosity, reflectiveness, and neutrality, qualities that are essential when working to facilitate system design groups (Hogan et al. 2014, 2015a, b; Broome and Hogan 2021). Curiosity implies maintaining an attitude of openness and interest to new ideas and lines of reasoning. Reflectiveness implies a questioning attitude to the potential ambiguity and redundancy of ideas, and the balance and soundness of arguments voiced as a group works to build systems models. Reflectiveness also implies the provision of feedback in relation to ideas and reasoning provided and the coordination of ideas and lines of reasoning to facilitate the integration of group members’ perspectives and contributions.

Reflective feedback is provided not only with an attitude of curiosity and openness, but also with neutrality as regards the underlying motives for particular ideas and lines of reasoning. In other words, the ideas and logic of group members are reflected upon by the facilitator based on core principles (e.g. clarity, non-redundancy, soundness) rather than on whether or not they fit with a particular political agenda or worldview.

At the same time, facilitators maintain awareness of the various political agendas and potential conflicts between group members that may need to be negotiated and managed during system design sessions. For example, Broome (2006) noted how gaining trust, maintaining impartiality, sustaining commitment, and dealing effectively with ongoing pressures to show ‘tangible’ results were critical in facilitating the systems thinking and design work supporting conflict resolution between Greek and Turkish Cypriots. In this sense, the facilitator role involves the exercise of skills that sustain effective team dynamics. Again, this implies the need to provide sufficient training for facilitators (Broome and Fulbright 1995).

## A Summary Overview of Our Current Pedagogical Focus

Over the past 7 years, we have worked at NUI, Galway to develop a dedicated applied systems science module, An Introduction to Collaborative Enquiry and Applied Systems Science. Our module builds upon Warfield’s (2006) work, by placing key tools and methods of Interactive Management (IM) at the core of the module, for example the nominal group technique (NGT) and interpretative structural modelling (ISM). Our module also incorporates the use of argument mapping, a tool which we have integrated with ISM to allow arguments within relational systems models to be mapped and analysed (Hogan et al. 2014). Finally, to support the translation of systems thinking into design solutions, students use scenario-based design (SBD) methods as part of their project work (Long et al. 2017).

Warfield’s (2006) approach to collaborative systems design is aligned with more recent approaches to computer-supported collaborative learning (CSCL) in the learning sciences (Stahl 2010, 2015). For example, Stahl (2015) highlights the need for development of curricula that incorporate a learning sciences emphasis on student-centred, collaborative, explorative, immersive, computer-supported problem-solving approaches. Also, Stahl’s (2010) conceptualisation of group cognition, places sequential small-group interaction as the central component, linking the task, the individual, and the group. This sequential small-group interaction, or ‘the dialogical interaction through which individual participants form into a collective knowledge-building agency’ (Stahl 2010: 255), is central to the development of shared understanding and learning at the group level. As such, consistent with both Warfield and Stahl, our PASSE learning activities are largely centred on small-group collaboration, in which group dialogue is facilitated by various instructional supports, tools, methodologies, and peer learning.

**Fig. 7:**
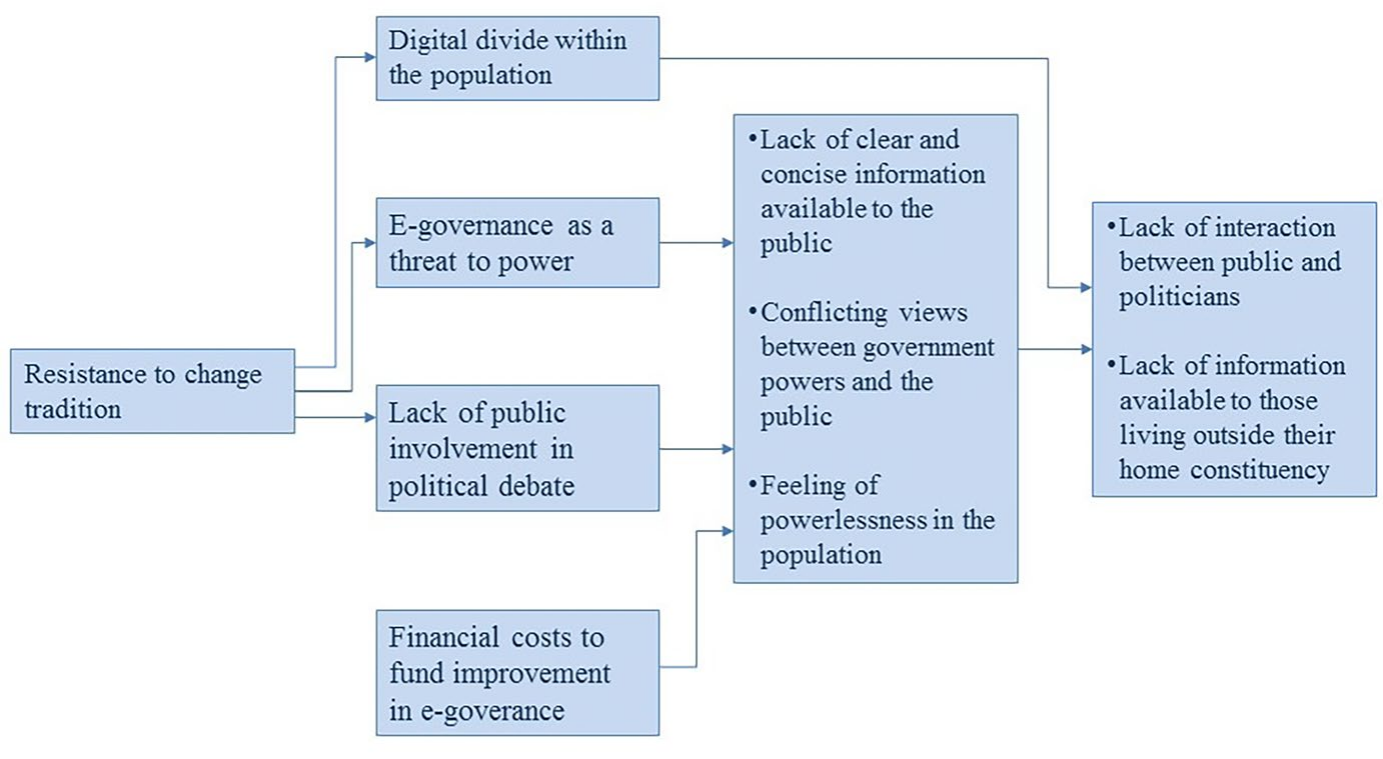
Sample ISM structure from a group project illustrating relationships between critical barriers to transparent, open democracy and government

To provide one example of an interpretative structural modelling (ISM) structure generated by students, Fig. 7 presents a systems model illustrating relations between barriers to transparent, open democracy and government.

The ISM structure presented in Fig. 7 is to be read from left to right, with arrows connecting boxes indicating ‘significantly aggravates’. Two or more barriers presented together in a box indicate that the barriers significantly, reciprocally aggravate one another. In the process of developing this model, students first used cooperative reading, literature review, and nominal group technique (NGT) to identify and clarify 41 barriers to transparent and open e-governance, before prioritising the 10 most critical barriers to be included in ISM structuring work.

Students identified resistance to change tradition as the primary driver of other barriers in the system of barriers. Resistance to change tradition was deemed to significantly aggravate the digital divide within the population, and the perception of e-governance as a threat to power, and lack of public involvement in political debate. Digital divide with the population, in turn, was argued to significantly aggravate both lack of interaction between public and politicians and lack of information available to those living outside of their home constituency.

Students went from here to developing options in response to the barriers in the field, and then in subsequent weeks they worked with specific scenarios to develop a set of user needs and mock-up design for a new open data e-government portal. They then presented their systems design work and mock-up design in an end-of-semester presentation. The overall week-by-week module focus is presented in Table 1.

The project work for this module culminates in the writing of a group project report, and individual student reflections on the collaborative system design process used in the module. Written feedback received to date suggests that students have responded well to the use of the tools and the structured approach to system design work, which involved following very specific steps in a group design process from week to week. For example, one student commented:

Particularly useful was the presence of a structure or plan that guided the group through the realisation of the project. This led the group to be more creative and relaxed, because the members knew what they needed to focus on at each stage. (Student 1)

Notably, this reflection is consistent with Soter and colleagues (2008), who propose that a constructive environment for collaborative discourse is one which provides structure and focus, while not being so rigid as to be prohibitive to generative learning.

Figure 7, depicting a systems model developed by one of the student groups, illustrates the engagement of students with systems thinking and the variety of relational judgements the group needed to consider during structuring. Generating a systems model of the relationships between barriers in a problematic situation is a demanding task. However, in their reflections, students highlighted the facilitation provided by instructors, as well as their own developing facilitation skills, as key to supporting this systems thinking process:

The quality of facilitation within the class was very high. During each discussion the instructor would join the group and add content, probe a specific question or ask for an evaluation of points being made. (Student 2)

**Fig. 8:**
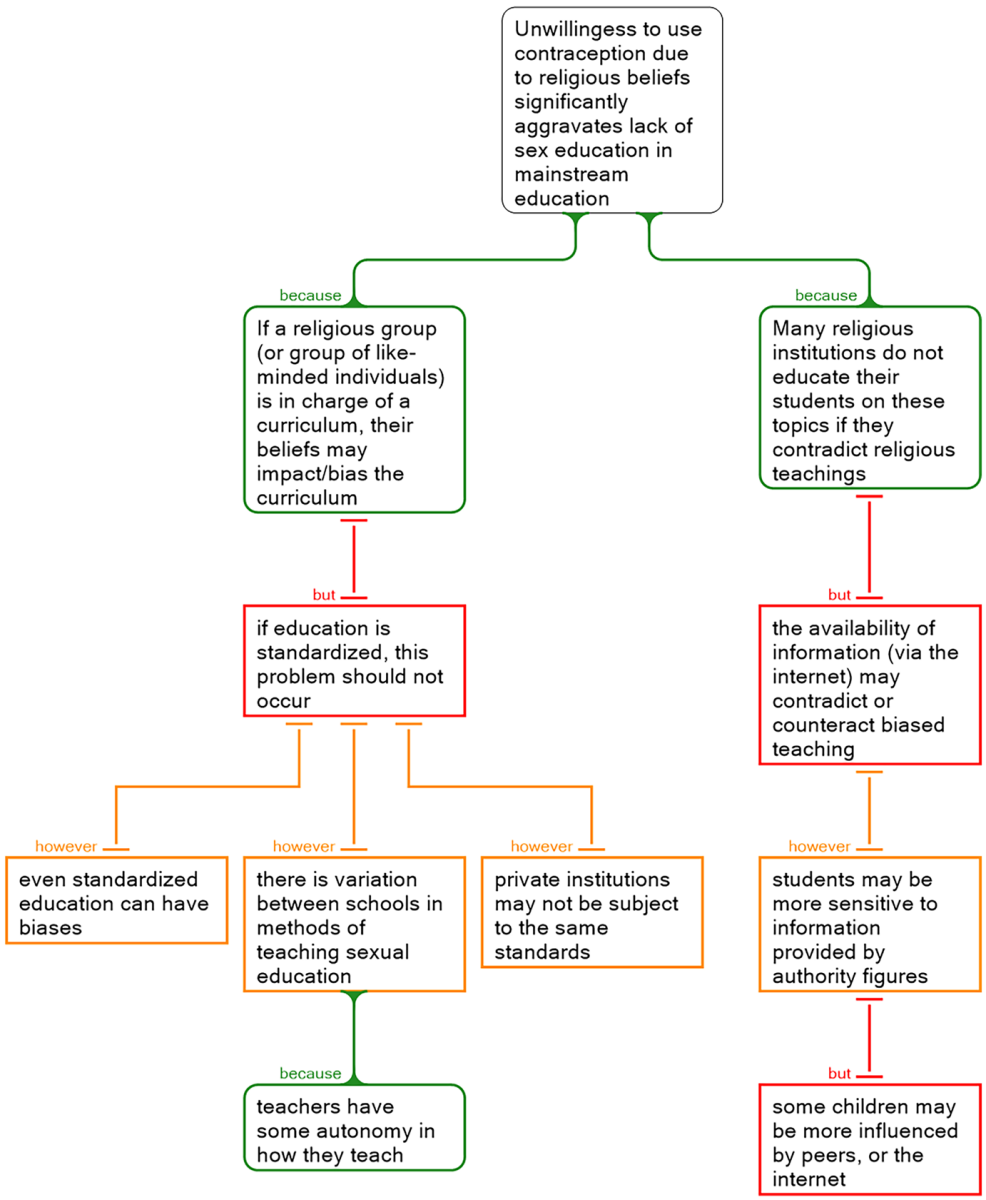
Sample argument map from a group project

The critical thinking skills employed by students during the process of working on their group project are reflected in the argument maps which they developed when thinking about key claims relevant to the problem they were addressing. For example, the argument map presented in Fig. 8 was used by a group to explore the topic of oral contraceptive pill usage, during their project on barriers to adherence to contraception. In the argument map presented below, students provide supports, objections, and rebuttals in relation to one relation (or claim) in their systems model.

A review of students’ weekly reflective journal entries, as well as their end of semester reflection on the collaborative process, suggests that positive group dynamics and effective teamwork featured strongly throughout their work together inside and outside of class, and developed during the semester. For example, a number of students drew contrasts between their experiences of group work in this module, versus previous experiences. For example:

I usually tend to avoid group projects, but I really enjoyed this group process…I haven’t really experienced that before in a group project. (Student 3)

Another student noted:

I think that the process encouraged the group’s teamwork abilities in that it provides us with new ways to communicate our ideas and structure our feedback. … I feel that this module has been an invaluable experience and has taught me many skills that can be applied to all of my future endeavours both as a student and possibly a practitioner. (Student 4)

##  Considerations for Future Iterations of PASSE

While the current iteration of our module represents a positive step towards the development of PASSE, a number of recommendations for future iterations of the module are proposed here.

First, consistent with Warfield’s (1976, 2006, 2010) focus on methodological rigour, future iterations of this applied systems science module should ensure to continually impress upon students the importance of methodology and process in collaborative problem-solving efforts. This reflects a deeper challenge to moderate intuitive, heuristic thinking and other sources bias and noise in both intra- and inter-individual cognitive action dynamics (Kahneman 2011; Kahneman et al. 2021).

The need to support and reinforce methodology in applied systems science applications is perhaps not surprising. Working with expert group facilitators, Broome and Fulbright (1995) found that methodology deficiencies were reported by experts as amongst the most influential barriers to effective group work. Broome and Fulbright also noted that many scholars have traditionally downplayed the importance of methodology in group problem-solving. However, as noted by Warfield (1976, 1995, 2004), there are a host of human cognitive and behavioral limitations that can be ameliorated through methodology, and the development of methodologies that support group problem-solving is very important for the future of PASSE.

Another recommendation for a future iteration of PASSE is based on a theme which emerged from students’ reflections. Given the significant and complex workload involved in the group projects, a number of students recommended that more time be devoted to the project in future (i.e. beginning the group projects earlier in the semester), to allow for more time at each stage of the systems thinking and design process (i.e. idea generation, categorisation, structuring, and scenario-based design). In this context, we have conceived of year-long PASSE programmes and also advanced postgraduate training programmes that include multiple complementary training components focused on skilled tool use, deeper reflection on group dynamics, and more intensive group facilitation training.

Each new module and programme iteration of PASSE will be unique in certain respects, in terms of group composition, problem focus, workspace, materials, time, and so on. Therefore, planning and simulation work needs to be done carefully in light of the specific teaching context, and plans need to be adapted as needed in light of constraints and unfolding group dynamics as the programme and curriculum is delivered.

As part of broader CI infrastructures (Mulgan 2018) and associated applied system science education design efforts (Hogan et al. 2017), further development of PASSE may be achieved through a cycle of iterations of design-based research (DBR). This will allow interventions to migrate from experimental classroom settings to average classrooms supported by realistic technological and personal support. DBR generally begins with an initial pilot designed to test an educational innovation (e.g. application of a systems thinking tool and methodology, implementation of a new module or programme), followed by assessments that help to further iterate the design and implementation. This may then be followed by a scaling-up of the pilot to a mainstream intervention for further development and evaluation, followed by a capstone intervention which seeks to further develop, fine-tune, verify, and consolidate its efficacy.

In advancing the design and delivery of PASSE, and empirically testing its efficacy, it would be beneficial for future iterations to measure key learning processes and outcomes during and after education, to assess learning gains, the development of collaborative competencies, and attitudes towards collaborative learning. Future iterations could incorporate objective measures of critical thinking, and systems thinking skills, as well as measuring changes team orientation, attitudes towards group learning, and other measures of teamwork.

By incorporating a broader transdisciplinary perspective and by building competence in applied system design in the context of productive relations between educational and research institutes, technology and business partners, and governments and citizens, postdigital applied systems science education may help to lead out in the further design of technology that can be used to enhance societal teamwork and system design capabilities that shape ongoing innovation and convergences arising from the Nano-Bio-Info-Cogno Paradigm. This is the consistent with the ways in which CINSU has contributed to supporting CI design work in the context of recent EU projects, and the ways in which CINSU project work has informed our developing PASSE perspective.

Importantly, in the PASSE framework presented in this paper, a postdigital orientation centres on a revolutionary focus on teamwork and empowerment of ethical CI design, with the fundamental ethics and ethos of groups focused on designs for sustainable wellbeing. PASSE is revolutionary in the sense that it entails a radical educational shift from a problematic and predominant focus on individuals to a focus on teams, systems, and collaborative designs for sustainable wellbeing (Hogan et al. 2014, 2015b; Hogan 2020; Hogan et al. 2021; Broome and Hogan 2021). Our teaching and learning efforts to date highlight some of the curricular and pedagogical challenges and possibilities.

## Conclusion

PASSE recognises the important relationships between education, science, and technology in addressing adaptive challenges central to the sustainability and flourishing of living and world systems. As part of human lifespan educational training, PASSE highlights the need to cultivate an understanding of systems, an understanding of group dynamics relevant to the management and design of systems, and skill in the application of applied systems science methods that can be used by groups in the management and redesign of systems. Although science and technology have expanded the diversity of knowledge, technical artefacts and solutions available to *Homo sapiens*, the societal foundation stone for the adaptive management of complex systems lags behind. In particular, there is little or no pedagogical focus on CI and applied systems science education (Broome and Hogan 2020; Hogan et al. 2017, 2018, 2021).

Without these foundations in education, and without a cultivation of collaborative and systems thinking capabilities, the political landscape is likely to become increasingly polarized and divisive and the cooperative relations between public and private sectors are likely to break down. As noted by Harari (2018) in *21 Lessons for the 21*^*st*^* Century*, while the grand unifying stories of twentieth century fascism (i.e. a world dominated by one group that subdues others) and communism (i.e. a world dominated by a centralised social system that ensures equality even at the price of freedom) have been largely abandoned, people have increasingly lost faith in the twenty-first century story of liberalism (i.e. a world in which humans cooperate freely and peacefully with minimal central control).

Harari (2018) observes how walls and firewalls between nations are increasingly visible, resistance to immigration is widespread, and the independence of the press and the judicial system is under threat. Many people see liberalisation and globalisation as a racket that has empowered a tiny elite and disempowered the vast majority, while peddling neoliberalism and individualism as a paltry philosophy to sustain resilience and wellbeing. Harari doesn’t assume to provide substantive solutions at the level of infrastructure design and indeed his ‘Lesson 19’ on education reinforces a focus on individual talents and does not highlight the inter-objective domain of group dynamics, teamwork, and CI system design. Indeed, by gravitating toward individual subjectivity (and meditation) in ‘Lesson 21’, Harari moves away from the inter-objective world and the associated challenges of collaborative system design.

We believe it is possible to respond to adaptive challenges with solidarity, CI, and coordinated, cooperative activity matched to the complexity of societal challenges (Hogan 2020; Mulgan 2018). Much like in other domains of our inter-objective world, our collaborative infrastructure design is open to innovation and transformation. Developments across the field of education, science, and technology are important in this regard. Importantly, scholars tracking developments across the biosciences, artificial intelligence, and education have characterised the emergence of a postdigital age, where digital technology is no longer separate from the natural and social world, and which no longer opposes the virtual world to the world of face-to-face experience, but rather sees the digital as increasingly integrated with our everyday actions and interactions (Jandrić et al. 2018; Knox 2019).

These convergences may appear inevitable on one level, but they are also open to design and can potentially reinforce teamwork, CI, and a more advanced understanding of systems supporting sustainable wellbeing. This view is consistent with our view that PASSE can promote the design and delivery of advanced educational infrastructures and pedagogies that foster the application of computer-supported collaborative systems design methods that allow us to address increasingly complex societal challenges. However, this requires a new framework focused on building teamwork, CI, and integral systems thinking and design skills as foundational to our educational and socio-political infrastructures and practices.

## Footnotes

1 For full details on IM methods and ways in which we have integrated IM with argument mapping and scenario-based design tools and methods, see Hogan et al. (2014, 2015b). Also, students taking our ASSE module work on projects connected with the work of our Collective Intelligence Network Support Unit (CINSU) at NUI, Galway. For more details on CINSU projects, see https://michaelhoganpsychology.com/collective-intelligence-network-support-unit-cinsu/. Accessed 23 February 2022.

2 One EU project we introduce to students is the ROUTE-TO-PA project—Raising Open and User-friendly Transparency-Enabling Technologies for Public Administrations. Central to the initial design work on this project was the use of collective intelligence methods to consult with stakeholders to identify requirements for a socio-technical infrastructure supporting citizens and public administrators working together to address shared problems using open data. One project area PASSE students are offered involves a focus on the design of new open data collaboration systems. In this context, they learn first about the ROUTE-TO-PA project. See https://www.routetopa.eu/. Accessed 9 March 2022.
